# Transcriptomic Analysis Reveals Diverse Expression of Scorpion Toxin Genes in *Mesobuthus martensii*

**DOI:** 10.3390/toxins16090399

**Published:** 2024-09-18

**Authors:** Zhongxian Yang, Haiquan Wang, Yan Zhao, Jianyu Huang, Chao Zhang, Zhiyong Di

**Affiliations:** 1 Key Laboratory of Zoological Systematics and Application of Hebei Province, School of Life Sciences, Hebei University, Baoding 071002, China; 2 Hebei Basic Science Center for Biotic Interaction, Hebei University, Baoding 071002, China

**Keywords:** *Mesobuthus martensii*, toxins, scorpion, transcriptome

## Abstract

Scorpions, an ancient group of venomous invertebrates, have existed for over 430 million years. Their toxins, important for predation and defense, exhibit a variety of biological and pharmacological activities. Research on scorpion toxins has spanned decades. Notably, the toxin genes of *Mesobuthus martensii* (Scorpiones: Buthidae), a well-known Chinese herbal medicine, have been described at genomic and proteomic levels. However, previous studies primarily focused on the toxin genes expressed in the venom glands, overlooking their expression in multiple tissues. This study analyzed transcriptomes from 14 tissues of *M*. *martensii*. Gene annotation revealed 83 toxin and toxin-like genes, including those affecting sodium, potassium, calcium, and chloride ion channels. Approximately 70% of toxin genes were highly expressed in the vesicle; additionally, some exhibited low or no expression in the vesicle while showing high expression in other tissues. Beyond the vesicle, high expression levels of toxin genes were observed in metasoma segments II-V, blood, lateral eyes, chelicerae, legs, pedipalp chelae, femurs, and patellae. This expression pattern suggests that toxin genes are recruited from multiple tissues and may help prevent intraspecific harm during courtship and competition for prey. These findings inspire further research into the evolutionary recruitment process of scorpion toxins.

## 1. Introduction

Scorpions are among the oldest terrestrial animal groups on Earth, found on all continents except Antarctica, demonstrating their strong adaptability to diverse environments. To date, approximately 2800 species of scorpions have been recorded across 232 genera and 23 families [1]. Scorpions originated in the sea and gradually migrated to land [2]. In Asia, *Mesobuthus martensii* is particularly notable for its widespread distribution, significant biomass, and extensive use in traditional Chinese medicine [3]. According to the “Combat poison with poison” strategy in traditional Chinese medicine, toxins from processed venomous scorpions have been used as a medicinal material in China for more than one thousand years [4]. These processed *Mesobuthus martensii* scorpions are called “Quanxie”, which are still widely used nowadays to treat various diseases according to the *Pharmacopoeia of the People’s Republic of China* [5]. Research on the toxins of *M*. *martensii* has been intense over recent decades, highlighting their value as medicinal resources. Cao et al. (2013) published the first complete genome of *M. martensii*, revealing over 32,016 genes, which include 116 neurotoxin genes: 61 NaTx (sodium channel toxins), 46 KTx (potassium channel toxins), 5 ClTx (chloride channel toxins), and 4 CaTx (calcium channel toxins) [6]. Of these, 51 NaTx genes and KTx genes were identified as clustered on 17 scaffold genes [6]. Subsequently, 153 fractions were isolated from the *M*. *martensii* venom using techniques such as 2-DE, SDS-PAGE, and RP-HPLC, and 227 non-redundant protein sequences were identified, including 134 previously known and 93 unknown proteins [7]. Among the 134 known proteins, 115 were first confirmed from *M*. *martensii* crude venom, with an additional 19 toxins verified, comprising 43 typical toxins, 7 atypical toxins, 12 venom enzymes, and 72 cell-associated proteins [7]. Furthermore, Di et al. (2022) identified four new NaTx genes in *M. martensii* [8]. These studies not only provide clues to understanding the molecular diversity of scorpion toxin peptides, but also provide a valuable resource of drug candidates for the treatment of ion channel diseases.

Venom is typically produced by venom glands; however, toxin genes have also been found to be expressed in non-venomous tissues of venomous animals. In the king cobra, toxin genes have been identified in a broad range of tissues [9]. These toxin genes are thought to have evolved from homologous genes with non-toxic functions, originally expressed in tissues lacking venom glands [10,11]. Zhao et al. (2018) detected the expression of toxin genes in the torso tissues of a centipede, demonstrating that the toxin genes in the venom gland originated from non-venomous tissues [12]. Mass spectrometry analysis of scorpion vesicle yielded 22 full-length and 44 truncated potassium channel toxins [13]. An analysis of scorpion tissue composition showed protein to be the main component, with the venom gland protein differing from other tissues [14]. Anti-tumor experiments on extracts from scorpion tail and torso tissues revealed that, under identical drug concentration conditions, tail extracts inhibited tumor growth more effectively than torso extracts [15]. This suggests scorpion toxin genes might also be expressed in non-venomous tissues, offering significant functional insights. 

Venomous animals serve as a crucial biological resource and have been utilized in Chinese medicine for over 1000 years. The discovery and application of venom peptides in pharmaceutical development have increased. However, research typically concentrates on scorpion venom, overlooking the potential of other tissues. This study explores the expression differences in toxin genes across various tissues through a transcriptome analysis of 14 tissues from *M*. *martensii*, uncovering the recruitment process of scorpion toxin genes and providing new perspectives on scorpion toxin gene research.

## 2. Results

Transcriptome sequencing of different tissues from *M. martensii* was conducted using the Illumina HiSeq™ 2000, which generated an average of 5.44 Gb from each sample. The raw reads totaled approximately 40–45 Mb. After the removal of low-quality, adaptor-polluted, and high-content unknown base (N) reads, clean reads ranged from 35 to 37 Mb. Following read filtering, HISAT [16] was used to map clean reads to the reference genome. An average of 49.71% of reads were mapped, and the uniformity of the mapping results suggested comparability across samples. After genome mapping, StringTie [17] was used to reconstruct the transcripts. Using genome annotation, we identified novel transcripts in our samples with cuffcompare (a tool of cufflinks) [18].

**Figure 1:**
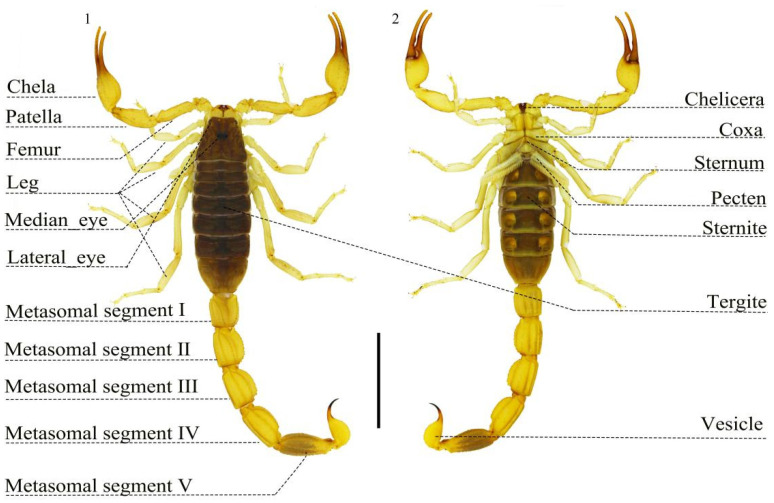
Dorsal (**left**) and ventral (**right**) views of *Mesobuthus martensii* (male).

To investigate the expression patterns of scorpion toxin genes across multiple tissues, transcriptome sequencing covered 14 tissues from *M. martensii*, including the blood, chelicera, coxa-sternum, lateral eye, leg, median eye, metasoma II-V, pecten, pedipalp chela, pedipalp femur, pedipalp patella, sternite, tergite, and vesicle. Scorpion venom is produced in the secretory epithelium of the two non-communicating venom glands located in the vesicle [19]. Supplementary Information S1 presents the FPKM of toxin genes from various *M. martensii* tissues. The morphological structures of *M. martensii* are illustrated in Figure 1, identifying 83 toxin genes, including putative new toxin genes. Of these, 57 recorded toxin genes (33 NaTx, 21 KTx, 2 ClTx, and 1 CaTx) and 26 new putative toxin genes were identified (Supplementary Information S2). In addition, we performed a multiple sequence alignment analysis of different toxin gene families (Supplementary Information S3). 

### 2.1. Expression of Sodium Channel Toxin Genes in Different Tissues

Sodium channel toxins are the main component of scorpion toxins [20]. They belong to the long-chain scorpion neurotoxin group, containing 58 to 76 amino acids, most with four pairs of disulfide bonds [21]. This study identified 39 NaTx genes, including 6 new putative members (BGI_novel_G002263, BGI_novel_G002900, BGI_novel_G003355, BGI_novel_G003788, MMa13213, and MMa44290).

**Figure 2:**
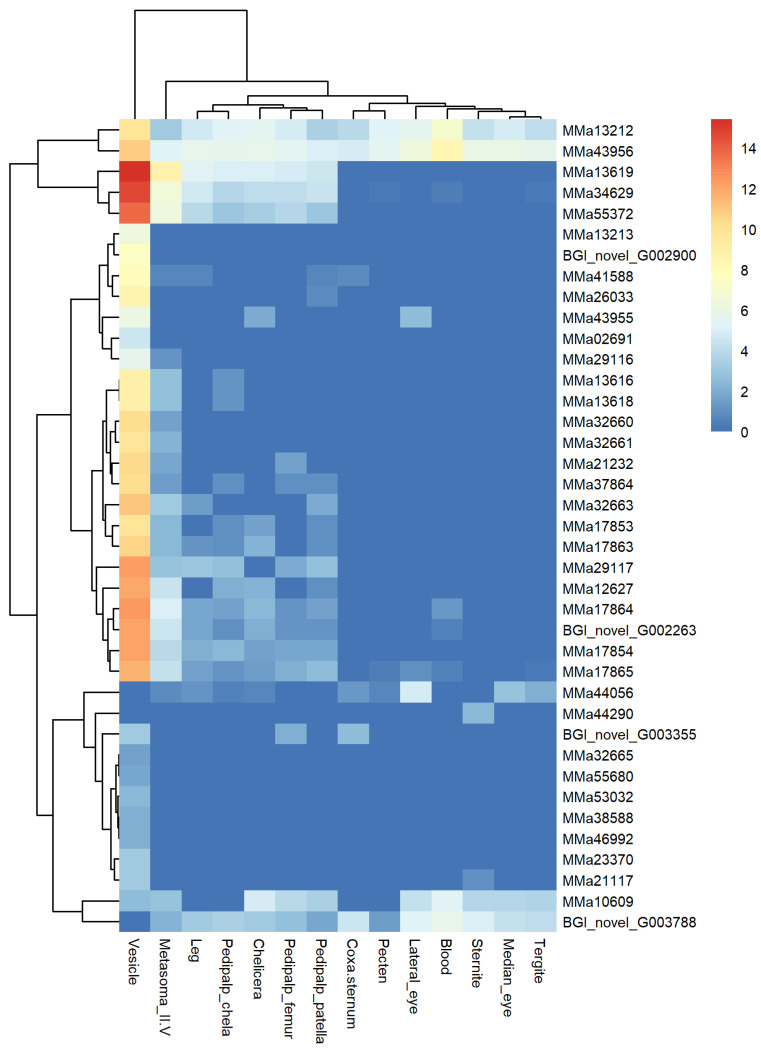
Heat map of 39 sodium channel toxin genes among 14 tissues of *Mesobuthus martensii.* The expression level is presented as log_2_(FPKM+1).

A heat map showed NaTx genes with similar expression patterns in the vesicle clustered together (Figure 2, Supplementary Information S1). Thirteen genes with no or low expression in the vesicle formed one subclade. Six toxin genes, MMa32665 (BmKNaTx13), MMa38588 (BmKNaTx65), MMa46992 (BmKNaTx28), MMa23370 (BmKNaTx44), MMa55680 (BmKNaTx54), and MMa53032 (BmKNaTx29), were expressed only in the vesicle, and two genes, BGI_novel_G003788 and MMa44056 (BmKNaTx25), were found in multiple tissues except in the vesicle. MMa44056 (BmKNaTx25) showed the highest expression in the lateral eye. BGI_novel_G003788 was highly expressed in blood and 13 other tissues excluding the vesicle. MMa44290 was expressed only in the sternite, and MMa10609, expressed in multiple tissues, showed the highest expression in the blood. Toxin genes expression in the vesicle were low, with FPKM values under 10; these genes were also lowly expressed in other tissues (FPKM < 60).

Toxin genes with significant expression in the vesicle were clustered in one subclade. Most NaTx genes in scorpions were expressed more in the vesicle than in other tissues, with 22 genes in this subclade. The FPKM values for these genes ranged from 20 to 5000. Genes like MMa13213 and MMa02691, with low expression levels, were only found in the vesicle. In contrast, MMa26033 (BmKNaTx10) and MMa2911 (BmKNaTx63) were lowly expressed in non-venomous tissues. Other genes, like MMa17864 (BmKNaTx5) and MMa17854 (BmKNaTx6), were highly expressed in the vesicle, with FPKM values over 1000. These genes shared a common expression feature in the chelicera, legs, metasoma_II-V, and pedipalp chela, femur, and patella.

The final subclade consisted of the three most highly expressed toxin genes and two genes expressed in all 14 tissues. The expression of MMa13619 (BmKNaTx3), MMa34629 (BmKNaTx64), and MMa55372 (BmKNaTx31) was similar to MMa17864 (BmKNaTx5) and MMa17854 (BmKNaTx6), but at higher levels, with a FPKM above 10,000. MMa13212 (BmKNaTx40) and MMa43956 (BmKNaTx23) were expressed in 14 tissue sites, highest in the vesicle and second in the blood.

### 2.2. Expression of Potassium Channel Toxin Genes in Different Tissues

Potassium channel toxins are widespread neurotoxins in scorpions, affecting various ion channels and fulfilling numerous biological functions [20,21]. Based on sequencing characteristics and function, these toxins were classified into five categories: α, β, γ, ξ, and κ [20,21]. This study identified 18 α-toxins, two β-toxins, one γ-toxin, and one putative potassium channel toxin. 

**Figure 3:**
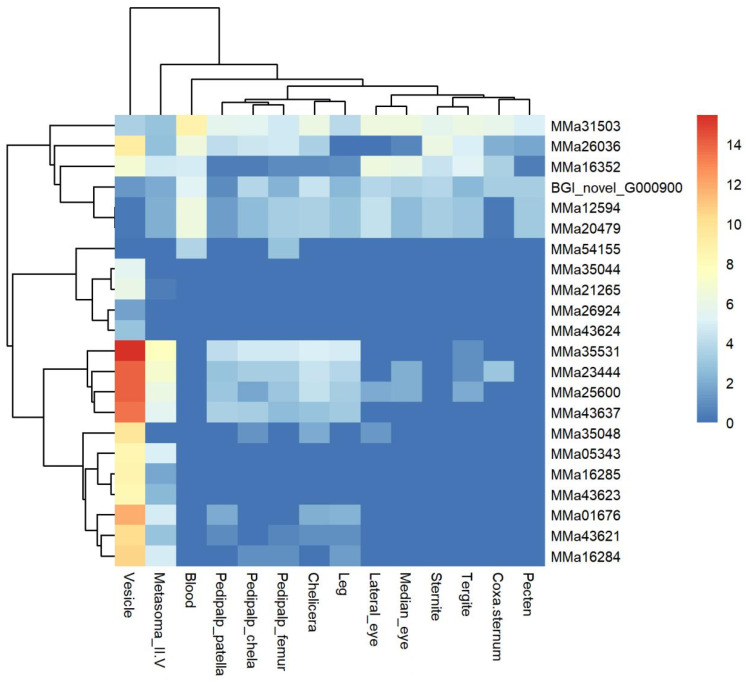
Heat map of 22 potassium channel toxin genes among 14 tissues of *Mesobuthus martensii.* The expression level was presented as log_2_(FPKM+1).

The heat map of the KTx genes indicated that the toxin genes most highly expressed in the vesicle formed a subclade (Figure 3, Supplementary Information S1). Thirteen KTx genes showed high expression in the vesicle, except for MMa26036 and MMa16352, which formed a different subclade due to their unique patterns. The FPKM values ranged from 100 to 40,000. MMa23444 (BmKaKTx28), MMa43637 (BmKbKTx1), MMa35531 (BmKbKTx2), and MMa25600 (BmKaKTx29) had FPKM values above 10,000, with MMa35531 (BmKbKTx2) showing the highest expression at 45,545.74 FPKM. These genes were also widely expressed in six other tissues: the chelicera, leg, metasoma_II-V, pedipalp chela, femur, and patella. This expression profile mirrored some NaTx genes.

Toxin genes expressed in multiple tissues formed a subclade with six genes. The expression level in this group was significantly lower than in the previously mentioned subclade. MMa26036 (BmKaKTx8) and MMa16352 (BmKaKTx27) showed the highest expression in the vesicle, while the other four genes were lowly or not expressed in the vesicle. MMa12594 (BmKaKTx36) and MMa20479 (BmKaKTx37) were expressed in all tissues except the vesicle and coxa-sternum. BGI_novel_G000900 was expressed in all tissues except the pedipalp patella. MMa31503 (BmKaKTx25) was expressed in 14 tissues, and these four genes had the highest expression levels in the blood.

Toxin genes expressed solely in one tissue formed another subclade. MMa26924 (BmKaKTx30), MMa43624 (BmKaKTx14), MMa35044 (BmKaKTx10), and MMa21265 (BmKaKTx4) were only expressed in the vesicle. MMa54155 (BmKrKTx2) was expressed only in the blood and pedipalp femur. In comparison, the gene expression level in this subclade was very low, with a maximum of 64.52 and a minimum of 1.98.

### 2.3. Expression of Calcium Channel Toxin Genes and Chloride Channel Toxin Genes in Different Tissues

**Figure 4:**
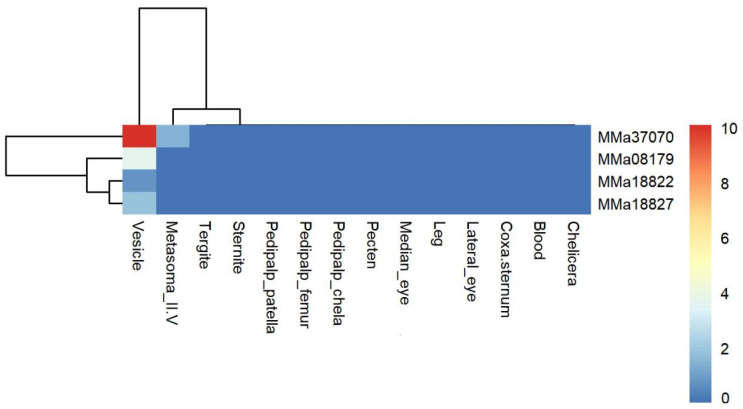
Heat map of 2 overlapping calcium channel toxin genes and 2 overlapping chloride channel toxin genes among 14 tissues of *Mesobuthus martensii.* The expression level is presented as log_2_(FPKM+1).

Both calcium and chloride channel toxins are components of scorpion venom. ClTx genes can block small-conductance chloride channels [22] and specifically act via inhibiting glioma invasion and metastasis [23]. CaTx genes regulate calcium influx into excitable cells, triggering physiological processes in vivo, such as nerve impulses, neurotransmitter release, opening downstream channels, muscle contraction, and gene expression [24]. Two ClTx genes, including one CaTx gene and a putative CaTx gene, were identified in this study. MMa08179 (BmKClTx5) showed expression solely in the vesicle with an FPKM value of 11.61. MMa37070 (BmKClTx1) was highly expressed in the vesicle (FPKM = 1094.39) and had low expression in Metasoma_II-V. MMa18822 (BmKCaTx4) and MMa18827 were micro-expressed only in the vesicle. Overall, ClTx genes exhibited higher expression levels than CaTx genes. Gene heat map clustering indicated that the ClTx gene (MMa37070) was separate from the other three CaTx toxin genes (Figure 4, Supplementary Information S1).

### 2.4. Expression of Toxin-like Genes in Different Tissues

In addition to ion channel toxins, numerous toxin-like and some toxin peptide genes were identified. Toxin-like genes were categorized into three types: U8-agatoxin-Ao1a-like, U24-ctenitoxin-Pn1a-like, and toxin CSTX-20-like. Venom peptide genes included astacin-like metalloproteases, and the function of some genes remained unknown (Supplementary Information S1).

U8-agatoxin-Ao1a-like

Agatoxins, chemically diverse spider toxins, were first isolated from *Agelenopsis aperta* [25]. These toxin genes are classified into different superfamilies based on cysteine motifs and target ion channels [26,27,28]. In this study, three genes were identified as U8-agatoxin-Ao1a-like, but none were expressed in the vesicle. The highest expression of MMa13545 and MMa15420 genes occurred in the coxa-sternum. The BGI_novel_G003302 gene was most highly expressed in the pecten and second-highest in the coxa-sternum.

U24-ctenitoxin-Pn1a-like

The ctenitoxin family contains two consecutive thyroglobulin type I repeat (TY) domains and a predicted signal peptide [29]. The function of the TY domain is proposed as a cysteine protease inhibitor. Eight scorpion toxin genes were annotated as U24-ctenitoxin-Pn1a-like genes. BGI_novel_G003352, MMa01529, MMa23846, and MMa47922 were not expressed in the vesicle and were most highly expressed in the lateral eye. BGI_novel_G003352 and MMa01529 were also abundantly expressed in other tissues. MMa53613 was expressed in all tissues except the vesicle, with the highest expression in the blood. MMa01531 was expressed in all tissues except the pedipalp patella, with the highest expression in the lateral eye. Ma17454 and MMa21474 were expressed in all tissues, with the lateral eye and blood being the highest expressive tissues for MMa17454 and MMa21474, respectively.

Astaxanthin-like metalloprotease

The astacin family of metalloproteases consists of zinc endopeptidases [30]. They participate in digestive functions, peptide processing, activating growth factors, degrading peptides, and processing extracellular molecules [31]. Besides toxin-like genes, three genes were annotated as astacin-like metalloproteases. MMa15230 was highly expressed in the vesicle and trace-expressed in other tissues. MMa17065 and MMa39677 were not expressed in the vesicle and were lowly expressed in other tissues, with the highest expressions in the lateral eye and sternite, respectively.

Based on the annotation results, four unknown toxin peptides were screened. BGI_novel_G002572, putatively an ion channel toxin, was expressed only in the vesicle. MMa13517, annotated as toxin CSTX-20-like, showed no expression in the vesicle but was widely expressed in the other 13 tissues, particularly in the lateral eyes. MMa29690, annotated as a toxin peptide, had the highest expression level in the vesicle. MMa11009, annotated as a toxin-like peptide, showed the highest expression in the blood.

**Figure 5:**
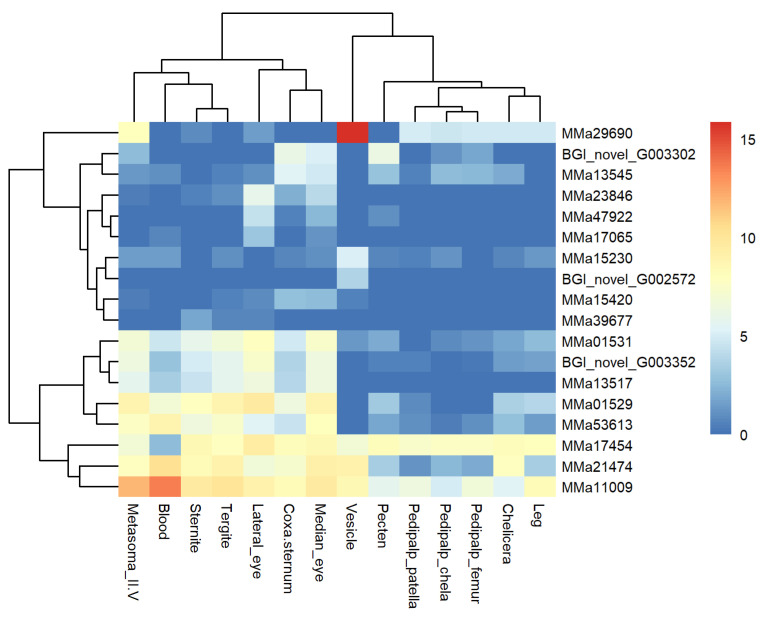
Heat map of 18 overlapping toxin-like genes among 14 tissues of *Mesobuthus martensii.* The expression level is presented as log_2_(FPKM+1).

According to the heat map, MMa11009 clustered with MMa21474, and MMa13517 clustered with BGI_novel_G003352 (Figure 5). We hypothesize that the two unknown-function genes MMa11009 and MMa13517 might perform a similar function to U24-ctenitoxin-Pn1a-like genes.

### 2.5. Validation of RNA-Seq

To verify the result, six tissues of different sexes were selected for transcriptome sequencing: the blood, chela, metasoma II, metasoma V, pecten, and vesicle. The scorpions used in the two rounds of RNA-seq were adult healthy individuals of the same species, and came from the same area. 

Some members of NaTx (MMa43956 and MMa10609), KTx (MMa35048 and BGI_novel_G000900), ClTx (MMa37070), CaTx (MMa18827), U8-agatoxin-Ao1a-like (BGI_novel_G003302), U24-ctenitoxin-Pn1a-like (MMa17454), and Astaxanthin-like metalloprotease (MMa15230) were selected to compare the expression difference between the two rounds of RNA-seq. MMa37070 and MMa18827 were not expressed in the second RNA-seq. Consequently, the Unigene13068-S7 (putative ClTx) and Unigene27593-S6 (putative CaTx), which exhibited the highest degree of sequence similarity with the aforementioned sequences, were selected as substitutes (Supplementary Information S2).

In the second RNA-seq, the expression patterns of MMa35048, BGI_novel_G003302, MMa17454, and MMa15230 in selected tissues remained unaltered, with only slight variations in FPKM (Figure S1). In the vesicle, the FPKM of MMa35048 in male samples was significantly higher than in female samples, approximately twice as much. The expression of MMa15230 in the vesicle was up-regulated compared to the first RNA-seq. In the second RNA-seq, the expression patterns of MMa43956 and BGI_novel_G000900 in selected tissues remained unaltered, but with significant differences in FPKM (Figure S1). The expression of MMa43956 was up-regulated compared to the first RNA-seq. The expression of BGI_novel_G000900 was down-regulated compared to the first RNA-seq. In the second RNA-seq, MMa10609 was also expressed in the pecten and chela, with up-regulated expression compared to the first RNA-seq (Figure S1). The expression patterns of MMa37070 and Unigene13068-S7, as well as MMa18827 and Unigene27593-S6, were identical in selected tissues. Additionally, in the vesicle, the FPKM of Unigene13068-S7 in female samples was significantly higher than in male samples, approximately twice as much (Figure S1). 

This analysis indicates that (i) the expression trends of the selected genes are essentially consistent between the two rounds of RNA-seq, with slight differences in expression levels; and (ii) the toxin genes are dynamically expressed, and these genes are not necessarily essential for the scorpion to perform normal physiological functions. Perhaps only a portion of the toxin genes expressed is enough, especially in tissues except the vesicle, where this dynamic expression is more evident. Furthermore, the expression levels of the selected genes in metasoma II, metasoma V, and metasoma II-V are essentially consistent. The expression levels of the selected genes in tissues from different genders are also essentially consistent; however, some genes show gender differences in vesicle expression levels. In summary, we consider the results of the first RNA-seq to be reliable.

## 3. Discussion

Scorpion venom, produced by the vesicle, is crucial in predation and defense against external predators [32]. Transcriptome sequencing analysis of 14 *M. martensii* tissues revealed higher toxin gene expression levels in the vesicle. Notably, certain genes were not expressed in the vesicle, whereas others showed relatively higher expression in non-venom-gland tissues. 

### 3.1. Toxin Genes Expressed Only in the Vesicle

A subset of ion channel toxin genes was expressed in the vesicle, including 12 NaTx, 4 KTx, 2 CaTx, 1 ClTx, and 1 ion channel toxin gene of unknown function. These genes exhibited a comparatively low expression level (FPKM < 100). We hypothesized that these vesicle-specific genes might encode core toxin effector proteins, similar to those in the Indian cobra [33]. 

### 3.2. Expression of Toxin Genes in Multiple Tissues (Highest Expression in the Vesicle)

Most ion channel toxin genes showed high expression in the vesicle (FPKM > 1000) and variable expression in 13 other tissues. These genes are notably present in multiple specific tissues, including the chelicera, leg, metasoma_II-V, pedipalp chela, femur, and patella. Similar expression patterns occur in other venomous species like centipedes [12], king cobras [9], Indian cobras [33], *Pteromalus puparum* [34], and spiders [35]. In king cobras, venom toxins originated from gene homologs with non-toxic functions that are not expressed in the venom gland [9]. In centipedes, toxin genes absent from the venom gland have undergone gene duplication, expanding the multilocus toxin gene families [12]. This supports the toxin recruitment hypothesis [36,37].

We propose hypotheses for the specific expression of scorpion ion channel toxin genes in multiple tissues, excluding the vesicle. Metasoma_II-V exhibited the highest expression levels besides the vesicle, potentially linked to the development of the scorpion metasoma. The evolution of the scorpion metasoma serves predatory and defensive roles [38], and metasomal segments develop later than the anterior appendages due to the anterior–posterior migration of the growth region [39,40]. Unlike anterior appendages, the venom gland in the metasomal segment is controlled by nerve endings from the fourth ganglion in the scorpion’s tail [41]. Therefore, we assume that the proximity of the metasomal segment to the venom gland and the similarity in their development lead to naturally high expression levels of toxin genes in these segments. 

**Figure 6:**
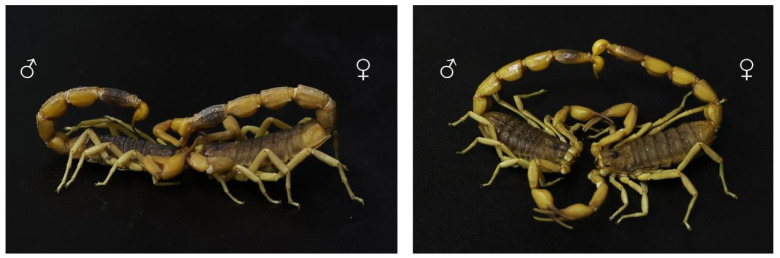
Intense sexual stinging behavior of *Mesobuthus martensii* during mating.

The tissues with high toxin gene expression levels include the terminal parts of scorpions’ metasomal segments. The behavior of scorpions inspired this phenomenon of expression (Figure 6). Scorpions exhibit sexual stinging behavior during the courtship mating phase. The male scorpion pierces the female with its aculeus, targeting the intersegmental membrane near the tentacular tibial segment. The aculeus remains in the female’s body for a period [42]. In courtship mating of *Scorpiops luridus*, the male stings the female’s first pair of legs or the pedipalp intersegmental membrane and remains there for about 120–300 s [43]. Venom might be injected during this process, and this stinging behavior may suppress the aggressive behavior of females during mating. Venomous animals exhibit significant tolerance to their toxins, including centipedes [44], poisonous frogs [45], and poisonous birds [46]. Some ion channels in *M. martensii* have been found to be insensitive to scorpion toxins [6]. Therefore, we hypothesized that toxin gene expression in these tissues may be related to tolerance to scorpion toxins.

### 3.3. The Expression of Toxin Genes in Multiple Tissues (No Expression or Relatively Low Expression in the Vesicle)

Some genes, annotated as potassium and sodium ion channel toxins and toxin-like genes, were found with no or low expression in vesicle. The low expression of these ion channel toxin genes in the vesicle may imply that they are not major components of scorpion venom. The toxin-like genes fell into three main categories: U8-agatoxin-Ao1a-like, U24-ctenitoxin-Pn1a-like, and Astacin-like metalloprotease toxin. Among them, the gene annotated as U8-agatoxin-Ao1a-like toxin showed almost no expression in the vesicle and low expression in other tissues. Some toxin genes annotated as U24-ctenitoxin-Pn1a-like were not expressed in the vesicle, while others were lowly expressed there. Most of these genes were highly expressed in the lateral eyes, except for MMa21474 and MMa53613. Toxin genes annotated as Astacin-like metalloprotease toxin MMa15230 had the highest expression level in the vesicle. MMa17065 had no expression in the vesicle and was lowly expressed in other tissues. The U8-agatoxin-Ao1a and U24-ctenitoxin-Pn1a genes derive from the spider venom gland [29,47]. Based on the expression characteristics of these three toxin gene homologs, we hypothesize that these toxin gene analogs are not the main components of scorpion toxins but are more likely to play roles in other physiological processes or have no function. 

Finally, it is necessary to acknowledge the limitations of this work. First, in this study, the number of *M. martensii* selected from different tissues during tissue extraction was inconsistent, which could potentially bias or limit the experimental results. In addition, because wild samples were selected from the same location in this study, genetic diversity among wild populations and expression differences in wild samples due to ecological factors were not examined, which may affect the generalizability of the experimental results.

## 4. Conclusions

The expression of scorpion toxin genes was achieved through RNA sequencing of 14 tissues. We found that scorpion toxin genes are expressed not only in the vesicle but also in multiple tissues, particularly in the blood, legs, chelicerae, lateral eyes, metasoma II-V, and pedipalp (chela, femur, patella). However, these tissues show no significant correlation with the production or function of the toxin genes. This finding suggests that the recruitment of scorpion toxin genes may originate from extra glandular tissues, which is inspiring for understanding the evolutionary recruitment of these genes. Whether the toxin genes serve specific physiological functions when expressed in extraglandular tissues remains a mystery. Future studies could provide important discoveries, offering new insights into the functional research of scorpion toxin genes.

## 5. Materials and Methods

### 5.1. Collection of Mesobuthus martensii

Healthy adult *M. martensii* individuals (both male and female) were collected from Xushui District, Baoding City, Hebei Province, China. The specimens were maintained temporarily at room temperature (about 25 °C) in the laboratory.

### 5.2. Tissues Collection

The number of male and female scorpions used for tissue extraction was nearly equal. Pooled scorpion tissues were used in this study, instead of individual or pool replicates. The numbers of adult individuals in each pool (transcriptome) were as follows—Vesicle: 15 males and females; Metasoma_II-V: 4 males and 4 females; Pedipalp_chela: 4 males and 4 females; Pedipalp_patella: 4 males and 4 females; Pedipalp_femur: 4 males and 4 females; Leg: 4 males and 4 females; Chelicera: 9 males and 7 females; Pecten: 9 males and 7 females; Lateral eye: 10 males and 10 females; Median_eye: 10 males and 10 females; Coxa-sternum: 4 males and 4 females; Tergite: 4 males and 4 females; Sternite: 4 males and 4 females; and Blood: 30 individuals in total. In order to collect enough tissues to complete the experiment, the number of *M. martensii* used in tissue collection was only related to the tissue size. The tissue collection for validation of RNA-Seq is the same as description above.

### 5.3. RNA Extraction and Sequencing 

Tissues intended for transcriptome sequencing were stored in liquid nitrogen immediately after collection. 

#### 5.3.1. RNA Extraction

RNA was extracted using Trizol as follows: Dispense 1.5 mL of TRIzol lysis buffer in a 2 mL tube. Take an appropriate amount of tissue samples, grind the tissue samples into powder with liquid nitrogen, and transfer into the lysis buffer. Stand flat for 5 min for the full lysis of tissue cells. Centrifuge the tissue samples after grinding and crushing at 12,000× *g* for 5 min at 4 °C. Transfer the supernatant to the centrifuge tube with 300 µL Chloroform/isoamyl alcohol (24:1) and mix thoroughly by upside-down violent shaking. Centrifuge at 12,000× *g* for 8 min at 4 °C. Transfer the supernatant to the 1.5 mL centrifuge tube, add 2/3 volume of isopropyl alcohol, gently invert and mix, and place in a −20 °C refrigerator for more than 2 h. Centrifuge at 17,500× *g* for 25 min in 4 °C. Discard the supernatant, wash with 0.9 mL 75% ethanol, and suspend the precipitation by inverting. Centrifuge at 17,500× *g* for 3 min at 4 °C. Discard the supernatant, centrifuge for a short time, use the tip to remove the residual liquid, and air-dry for 3–5 min. Dissolve the precipitation with RNase-free water.

#### 5.3.2. mRNA Library Preparation and Sequencing

A certain amount of RNA samples were denatured at a suitable temperature to open their secondary structure, and mRNA was enriched by oligo (dT)-attached magnetic beads; the reaction system was configured. After reacting at the suitable temperature for a fixed period of time, RNAs were fragmented as follows by preparing the first-strand synthesis reaction system, setting up the reaction program, synthesizing the first-strand cDNA, preparing the second-strand synthesis reaction system, and setting up the reaction program to synthesize the second-strand cDNA. After the reaction system and program were configured and set up, double-stranded cDNA fragments were subjected to end repair, and then a single ‘A’ nucleotide was added to the 3′ ends of the blunt fragments. The reaction system and program for adaptor ligation were subsequently configured and set up to ligate adaptors with the cDNAs. The PCR reaction system and program were configured and set up to amplify the product. Single-stranded PCR products were produced via denaturation. The reaction system and program for circularization were subsequently configured and set up. Single-stranded cyclized products were produced, while uncyclized linear DNA molecules were digested. Single-stranded circle DNA molecules were replicated via rolling cycle amplification, and a DNA nanoball (DNB) which contained multiple copies of DNA was generated. Sufficient quality DNBs were then loaded into patterned nanoarrays using a high-intensity DNA nanochip technique and sequenced through combinatorial Probe-Anchor Synthesis (cPAS). The cDNAs from various tissues were sequenced using the Illumina HiSeq™2000 platform. 

### 5.4. Data and Comparative Expression Analysis

The sequenced raw data were filtered by SOAPnuke (v1.5.2) to remove joint contamination and low-quality reads [48] (parameters: –n 0.01 –l 15 –q 0.4 –G). The clean data comparison to the reference genome sequence was performed using HISAT (v2.1.0) (parameters: –sensitive –no-discordant –no-mixed –I 1 –X 1000 –p 8 –rna-strandness RF). Clean data were compared to the reference gene sequence using Bowtie2 (v2.2.5) [49] (parameters: –q –sensitive –dpad 0 –gbar 99999999 –mp 1,1 –np 1 –score-min L,0, –0.1 –I 1 –X 1000 –no-mixed –no-discordant –p 8 –k 20 –N 1), and the gene expression levels for each sample were calculated using the RESM (v1.2.8) software package [50] (parameters: default). Genes were defined as expressed if they met the criterion FPKM ≥ 1 in any population. Unless otherwise specified, expression levels in this analysis are presented as FPKM. All genes were annotated using BLASTX and searched against protein databases (NR, SwissProt), and annotated with given descriptions, with the cutoff set at 1 × 10^−5^. Based on the annotation information, 83 toxin genes were identified and named according to the discovery and verification information of *M. martensii* toxin genes provided by Cao et al. (2013) [6]. The reference genome ID is available via the National Center of Biotechnology Information (https://www.ncbi.nlm.nih.gov/datasets/genome/GCA_000484575.1/; last accessed 4 September 2024). Differential gene detection results were used for hierarchical cluster analysis of concatenated differential genes using the Pheatmap package in R (v4.3.2). Data were standardized using log_2_(FPKM+1). Multiple sequence alignment of toxin gene families was performed using CLUSTAL W (v2.1).

## Data Availability

Data are contained within the article and Supplementary Materials.

## Supplementary Materials

The following supporting information can be downloaded at: https://www.mdpi.com/article/10.3390/toxins16090399/s1, Supplementary Information S1: The FPKM of 83 toxin and toxin-like genes of different tissues in the gene expression of *M. martensii,* including gene annotation results. Supplementary Information S2: The amino acid sequence of toxin genes. Supplementary Information S3: The results of the multiple sequence alignment of toxin gene families. Figure S1: The expression of toxin genes in selected tissues.
