# Supplementary figures and images for: Transcriptomic Analysis Reveals Diverse Expression of Scorpion Toxin Genes in Mesobuthus martensii
Source: Toxins (Basel). 2024 Sep 18;16(9):399. doi: 10.3390/toxins16090399 (PMC11435589; doi:10.3390/toxins16090399)

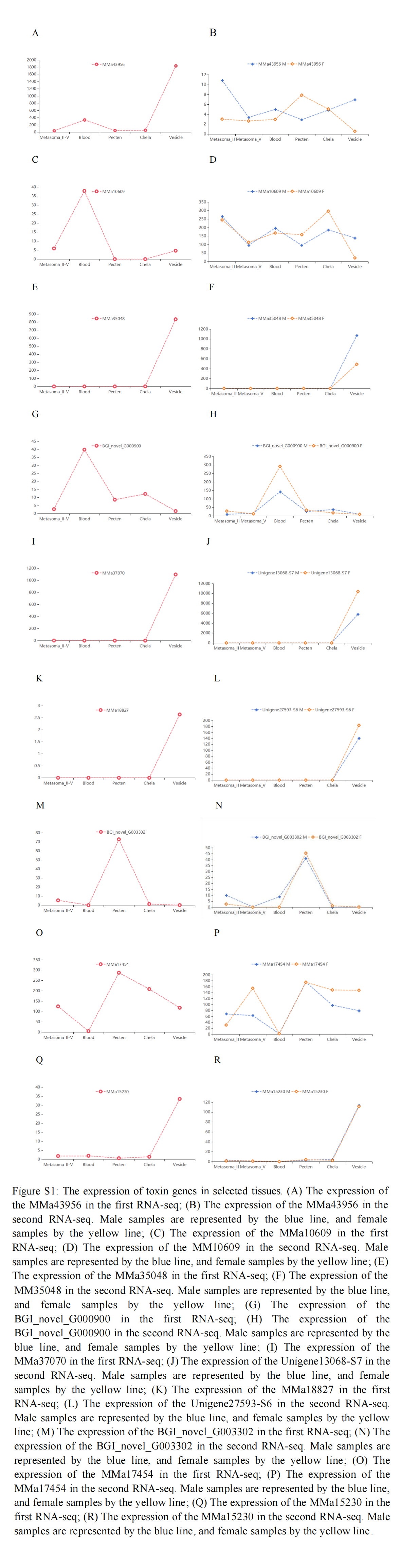

Supplement: Supplementary file 1 [file toxins-16-00399-s001.zip › Figure S1.png]
